# Design and Synthesis of Metal Complexes of (2*E*)-2-[(2*E*)-3-Phenylprop-2-en-1-ylidene]hydrazinecarbothioamide and Their Photocatalytic Degradation of Methylene Blue

**DOI:** 10.1155/2013/828313

**Published:** 2013-12-02

**Authors:** P. Murali Krishna, N. B. Gopal Reddy, Nagaraju Kottam, B. C. Yallur, Hussain Reddy Katreddi

**Affiliations:** ^1^Department of Chemistry, M. S. Ramaiah Institute of Technology, Bangalore 560 054, India; ^2^Department of Chemistry, Sri Krishnadevaraya University, Anantapur 515 006, India

## Abstract

The photocatalytic degradation has been considered to be an efficient process for the degradation of organic pollutants, which are present in the effluents released by industries. The photocatalytic bleaching of cationic dye methylene blue was carried out spectrometrically on irradiation of UV light using Cu(II), Ni(II), and Co(II) complexes of (2*E*)-2-[(2*E*)-3-phenylprop-2-en-1-ylidene]hydrazinecarbothioamide (HL). The effects of pH and metal ion were studied on the efficiency of the reaction. Cu(II) complex shows better catalytic activity and the highest percentage degradation (~88.8%) of methylene blue was observed at pH 12. A tentative mechanism has also been proposed for the photocatalytic degradation of methylene blue.

## 1. Introduction

Water pollution can be defined as any physical, chemical, or biological alteration in water quality that affects living organisms. Industries and human communities are the main sources, which mainly caused the pollution of surface waters like rivers, lakes, and seas [1]. Dye is a common contaminant or pollutant compound that can be easily found in wastewater [2]. Many industries are using dyes to colour their products, and they consume substantial volumes of water. The presence of small amounts of dyes in water is highly visible and undesirable [3]. Dyes are mainly applied in textile manufacturing, leather tanning, paper production, and food technology industries. The discharge of the major constituent of pollutants in wastewater into water sources from textile and other industries affects water quality including temperature, turbidity, pH, alkalinity, acidity BOD, COD, and colour. The discharge of those coloured wastewaters in the ecosystems causes serious environmental problems like aesthetic pollution and eutrophication and can originate dangerous by-products through oxidation, hydrolysis, or other chemical reactions in the wastewater phase [4, 5]. Therefore, the wastewater treatment is desirable to overcome this problem. Methylene blue is a kind of cationic dye (basic dye) containing heterocyclic aromatic chemical compound with the molecular formula C_16_H_18_N_3_SCl. This dye is generally classified as a basic dye and cationic species due to the presence of positively charged quaternary nitrogen atoms (as NR_3_^+^ or = NR_2_^+^). These groups enhance the solubility of dye in water due to their ionic characteristic [6]. Several methods of treatment of effluents have been undertaken from time to time; the most common methods are chemical precipitation and biological methods. However, these methods suffer from many disadvantages, but photocatalytic degradation seems to be the most promising advanced oxidation processes (AOPs) since it is of low cost and less time consuming [7] and is based on the generation of hydroxyl radicals [8, 9], which can be used for nonspecific oxidation of a wide range of organic compounds. AOPs include the classical Fenton reaction as well as its modifications (e.g., light assisted Fenton oxidation or ferrioxalate-photo Fenton oxidation) as well as H_2_O_2_/UV or ozonization [10–14]. However, Fenton's reagent can work only under acidic conditions (pH 2–4), and some dyes are not completely decomposed [15, 16]. Another set of AOP reactions makes use of transition metal ions (mostly Cu) in combination with ligand molecules for the decomposition of hydrogen peroxide and the resulting production of hydroxyl radicals. The main advantage of such systems is the broad substrate specificity and the ability to perform at pH 3–9 [16, 17]. Copper(II) complex systems have been used for the degradation of lignin [18], polycyclic aromatic hydrocarbons [19], and synthetic dyes [17, 20]. Hence, researchers are keen to search for a low-molecular weight ligand with high decolonization efficiency to be used in these reactions and to demonstrate the involvement of hydroxyl radicals in the decolonization reaction. The literature survey reveals that the metal complexes used for the degradation of dyes are nitrogen and oxygen containing copper complexes, and we are not aware of the literature about the usage of copper, nickel, and cobalt complexes containing sulphur and nitrogen as donor atoms. Herein, we report for the first time the results of our attempts to reduce/remove the methylene blue dye from sewage water using copper(II), nickel(II), and cobalt(II) complexes of (2*E*)-2-[(2*E*)-3-phenylprop-2-en-1-ylidene]hydrazinecarbothioamide.

## 2. Experimental

### 2.1. Materials and Methods

All the reagents used were of analytical grade and used without further purification. Thiosemicarbazide and (2*E*)-3-phenylprop-2-enal were purchased from Sigma-Aldrich, Bangalore, India. Metal chlorides were purchased from Merck. Methylene blue (MB) was purchased from S D Fine Chem. Limited, Mumbai. All the solvents were used as supplied without further purification.

The ligands and complexes were analyzed for their C, H, and N estimated on Perkin-Elmer 2400 CHN elemental analyser. Infrared spectra were recorded in the 4000–400 cm^−1^ region (KBr disc) on a Nicolet protege 460 FT-IR spectrophotometer. The molar conductance (10^−3^) in DMF at 30  ±  2°C was measured with a CC180 model (ELICO) direct reading conductivity bridge. Magnetic susceptibility measurements were made for all the complexes at 298 K using magnetic susceptibility balance (Sherwood Scientific, Cambridge, UK). High purity hydrated copper sulphate was used as a standard.

### 2.2. Preparations of Ligand (HL)

**Scheme 1:**
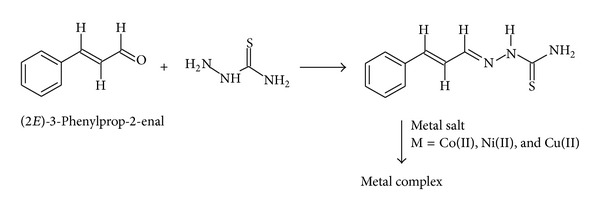
Synthesis of ligand and its complexes.

The ligand was prepared by condensing thiosemicarbazide with (2*E*)-3-phenylprop-2-enal by following the general procedure [21] given in Scheme 1. To a 5% glacial acetic acids in water solution (20 mL) of thiosemicarbazide (1 mol), add ethanolic solution (10 mL) of (2*E*)-3-phenylprop-2-enal (1 mol) the mixture was refluxed for 2-3 hrs. The crystalline product was collected by filtration, washed several times with hot water, and dried. All the thiosemicarbazones were recrystallised from ethanol.

### 2.3. Preparation of Complexes

The synthesis of complexes was based on the general procedure. To a solution of Ligand (HL) in 15 mL of methanol, appropriate metal chlorides were added and refluxed for 3 hrs. The formed precipitate was then filtered off, washed with cold methanol, and dried.

### 2.4. Photocatalytic Activity

**Figure 1:**
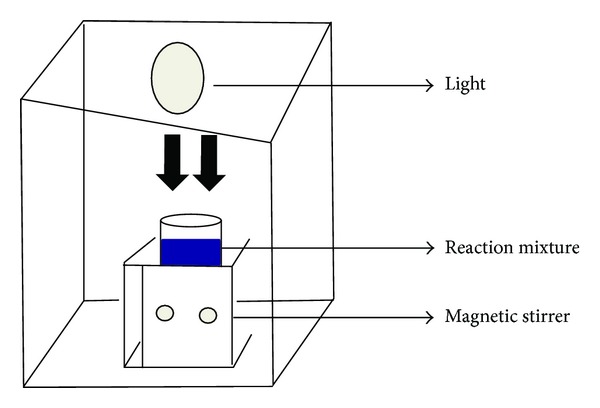
Photocatalytic experimental setup.

A photocatalytic activity of metal complexes (MC) on the degradation of methylene blue (MB) in water was examined with and without UV-light (Figure 1). A 300 W high pressure mercury-vapour lamp (*λ* < 380 nm) is a light source. It is well known that the adsorption and degradation of methylene blue in aqueous solution by the catalyst take place simultaneously. Hence, the effect of aqueous pH on the adsorption and degradation of methylene blue was investigated at the pH values of 3, 8.2, and 12, respectively. Then, the percentage degradation of dye was calculated using the following equation:

(1) $$\begin{array}{c} \text{Percentage}\;\text{Degradation}\;\left(\%\right)=\left[\frac{\left(A_{0}-A\right)}{A_{0}}\right]\times 100, \end{array}$$

where *A*_0_ and *A* are the initial and final concentration of the dye. The initial and final concentration of the dye were measured at regular intervals using a UV-visible spectrophotometer.

## 3. Results and Discussion

### 3.1. Analytical Data and Magnetic Moments

All the three complexes were found to be air stable and readily soluble in aprotic solvents such as DMF and DMSO due to the substituted groups which increase repulsion and thereby reduce the aggregation of molecules. The solubility decreases in strongly polar solvents such as methanol, ethanol, and water because the alkyl groups and other substituted groups are lipophilic and weakly polar [22]. The conductivity data suggest nonelectrolytic nature of complexes [23]. Room temperature magnetic moment data (Table 1) of the complexes suggest that the complexes were monomeric in nature and that the metal-metal interaction was absent.

### 3.2. Infrared Spectra

The IR spectra of the complexes are compared with the ligand spectra. Important infrared spectral data and their tentative assignments are presented in Table 2. The band assigned to (C=N) at 1621 cm^−1^ in the spectrum of the ligand shifts to 1567–1615 cm^−1^ in the spectra of complexes suggesting coordination of the imine nitrogen. Coordination of azomethine nitrogen is confirmed with the presence of new band at 355–443 cm^−1^, assignable to (M–N) [24, 25]. The IR spectrum of free ligand exhibits a medium band at 3153 cm^−1^, which is assigned to (N–H) vibration. The absence of (N–H) band in the spectrum of complex provides a strong evidence for the ligand coordination around metal ion in its deprotonated form in nickel complex (except for copper and cobalt complexes). The absorption attributed to (C=S) at 1178 cm^−1^ in the spectra of the free ligand shifts to 1159 cm^−1^ in the spectra of the copper complex, indicating coordination through a thioketonic sulphur, whereas in Ni(II) complex the disappearance of (C=S) and the presence of new band at 677 cm^−1^ indicate the coordination of thiolate sulphur [26] and also the presence of (M–S) band at 472 cm^−1^ [27, 28].

### 3.3. Electronic Spectra

The electronic spectra of the complexes are recorded in DMSO solvent and are given in Table 1. The electronic spectra of copper complex show charge transfer bands in high energy region and d-d bands in low energy region. The d-d band is assigned to ^2^E_g_ → ^2^T_2g_ transition suggesting distorted octahedral structure with moderate Jahn-Teller effect. But in the case of nickel(II) and cobalt(II) complexes, three bands are observed. These bands are assigned to the following transitions in the favour of octahedral structure:

 for Nickel(II) complex: ^3^A_2g_(F) → ^3^T_1g_(F), ^3^A_2g_(F) → ^3^T_1g_(F) and A_2g_(F) → ^3^T_1g_(F),
 for Co(II) complex: ^4^T_1g_ → ^4^T_2g_, ^4^T_1g_(F) → ^4^A_2g_(F) and ^4^T_1g_(F) → ^4^T_1g_(P).

**Figure 2:**
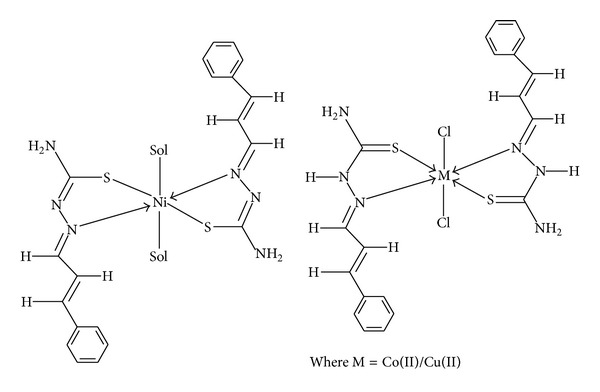
Tentative structures of copper(II), Ni(II), and Co(II) complexes.

Based on the analytical data and spectral data, tentative structures of the complexes are shown in Figure 2.

### 3.4. Photocatalytic Activity of Metal Complexes

**Figure 3:**
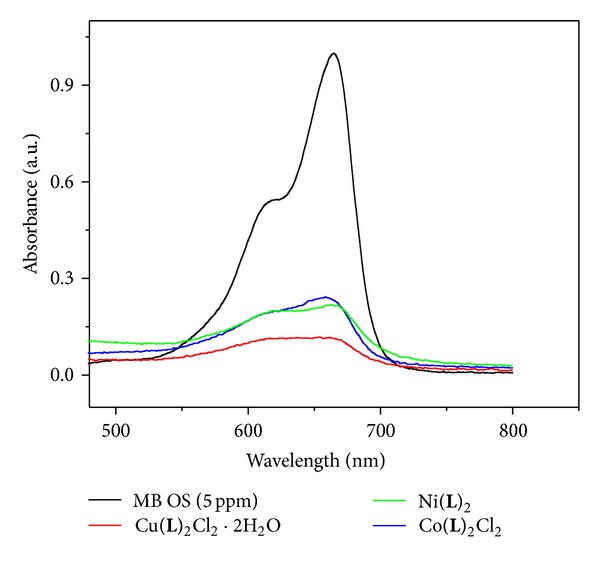
Degradation of methylene blue under UV light.

**Figure 4:**
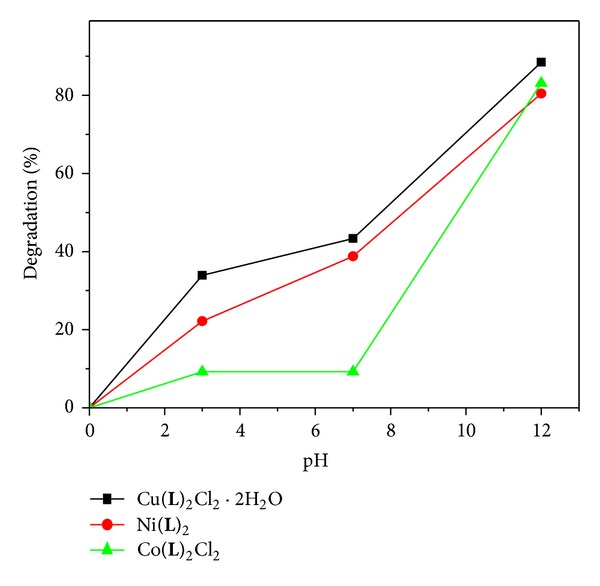
% degradation of methylene blue at different pH conditions.

The activities of the catalysts were evaluated in the photodegradation of MB at room temperature. 100 mg of MC was added into 100 mL of aqueous MB solution (5 mg/L), and the solution was stirred for about 15 min. The solution was then exposed to UV light at a distance of 4-5 cm between the liquid surface and the lamp. The solution kept stirring during irradiations. Photocatalytic degradation using different MCs viz copper, nickel, and cobalt complexes was studied (Figure 3). Experimental results show that copper complex shows maximum degradation at 12 pH (Figure 4). The observed high photocatalytic activity is awkward due to the delocalization of electrons in the conjugated MC as the photocatalytic effect depends on the enhancement in electron-hole (e^−^/h^+^) separation [29].

#### 3.4.1. The Effect of Solution pH

The effect of pH on the adsorption of methylene blue on metal complex has been studied by selecting the pHs 3, 8.2, and 12. The results are summarized in Table 3. In general, the photochemical degradation depends strongly on the pH of the reaction medium and the same is observed in the present study. The uptake of methylene blue increased rapidly with the increase of pH. The dye degradation is more favorable at higher pH condition due to the formation of more OH radicals, which will be utilized for oxidative degradation of methyl blue. A similar investigation reported that the adsorption of dye increased by increasing the solution pH [7].

**Figure 5:**
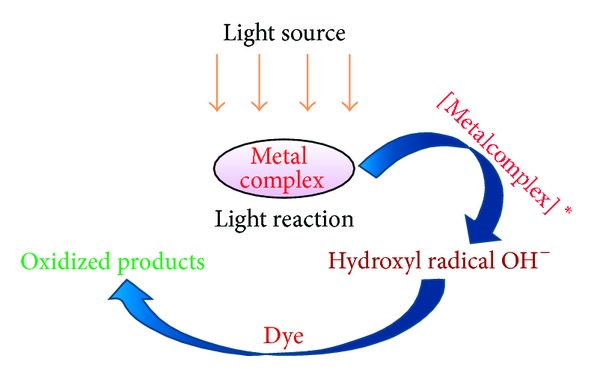
Photodegradation mechanism of metal complex.

*Mechanism*. Metal complex system produced hydroxyl radicals during the catalytic reaction. This is not surprising, since the production of –OH was already confirmed in other metal-based AOP systems [17, 30]. The radicals are strong oxidizing agents that react with dyes and cause their decolorization. The tentative mechanism has been proposed for the degradation of methylene blue in the presence of copper, nickel, and cobalt complexes and light (Figure 5).

Consider

(2) [MLn]+Light→[MLn]∗+O32→OH•OH•+Dye→Oxidative  products(M  stands  for  copper,nickel,and  cobalt).

## 4. Conclusions

Co(II), Ni(II), and Cu(II) complexes of (2*E*)-2-[(2*E*)-3-phenylprop-2-en-1-ylidene]hydrazinecarbothioamide (HL) are synthesized and characterized based on the various spectroscopic techniques. The rate of photocatalytic degradation of methylene blue using prepared metal complexes was successfully carried out under UV light. The increasing order of the rate with different metal complexes is as follows: Cu(II)  >  Co(III)  >  Ni(II). The generation of the hydroxyl radical is responsible for the degradation of dye photocatalytically. The dye has been successfully degraded 88.8% in the presence of copper complex in UV light at pH 12.

## Figures and Tables

**Table 1:** Analytical data of ligands and their complexes.

Complexes	Yield (%)	M.P (°C)	Elemental analysis found (Cal.)				*μ*_eff_	Molar conductance	Electronic spectral data	
			Carbon	Hydrogen	Nitrogen	Sulphur	(BM)		Electronic transition	Assignment
[**HL**·H_2_O]	90	114-115	58.41 (58.50)	5.20 (5.40)	20.49 (20.48)	15.51 (15.62)	—	—	—	—
[Co(**L**)_2_Cl_2_]	75	116–118	44.08 (44.45)	4.20 (4.10)	15.14 (15.55)	15.36 (11.87)	1.79	6	8710 (20.3)	^4^T_1g_→^4^T_2g_,
									15385 (14.7)	^4^T_1g_(F)→^4^A_2g_(F)
									29412 (151.2)	^4^T_1g_(F)→^4^T_1g_(P)
[Ni(**L**)_2_]	80	255-256	52.26 (51.42)	4.82 (4.64)	17.88 (17.98)	13.85 (13.73)	3.39	15	12658	^3^A_2g_(F)→^3^T_1g_(F)
									21505	^3^A_2g_(F)→T_1g_(F)
									28985	^3^A_2g_(F)→^3^T_1g_(F)
[Cu(**L**)_2_Cl_2_·2H_2_O]	72	185–188	41.60 (41.34)	4.23 (4.51)	14.45 (14.46)	11.20 (11.03)	1.58	10	12658	d-d band charge transfer band
									22222	

**Table 2:** Infrared vibrational bands (cm^−1^) of ligands and their complexes with tentative assignment.

Ligand/complex	*υ* (NH_2_)	*υ* (NH)	*υ* (C=N)	*υ* (C=S)	*υ* (C–S)	*υ* (M–N)	*υ* (M–S)
**HL**.H_2_O	3417 (br), 3261 (s)	3153 (br)	1621 (br)	1178 (s)	—	—	
[Co(**L**)_2_Cl_2_]	3416 (br), 3261 (s)	3154 (br)	1610 (br)	—	—	443 (s)	—
[Ni(**L**)_2_]	3426 (br), 3228 (s)	—	1567 (s)	—	677	355 (s)	472
[Cu(**L**)_2_Cl_2_·2H_2_O]	3463 (br), 3211 (s)	3050 (br)	1615 (br)	1159 (s)	—	—	

**Table 3:** Decolorization of dyes using Cu(II), Ni(II), and Co(II) complexes.

Dye/metal complex (5 mg)	% of removal at pH's without UV			% of removal at pH's with UV		
	3.0	8.2	12.0	3.0	8.2	12.0
Methylene blue	No change	13.82	24.0	5.69	29.93	47.73
Co(**L**)_2_Cl_2_	14.78	14.78	44.66	9.24	9.24	83.07
Ni(**L**)_2_	7.9	29.93	35.72	38.8	22.18	80.44
Cu(**L**)_2_Cl_2_·2H_2_O	33.92	8.83	33.44	43.33	3.3	88.80
